# Molecular Mechanisms Involved in the Aging of the T-cell Immune Response

**DOI:** 10.2174/138920212803759749

**Published:** 2012-12

**Authors:** Marco Antonio Moro-García, Rebeca Alonso-Arias, Carlos López-Larrea

**Affiliations:** 1 Immunology Department, Hospital Universitario Central de Asturias, 33006-Oviedo, Spain; 2 Fundación Renal “Iñigo Alvarez de Toledo”, Madrid, Spain

**Keywords:** Differentiation, Epigenetic regulation, Immunosenescence, T-cell signalling, Telomere shortening, Thymic involution, T-lymphocytes.

## Abstract

T-lymphocytes play a central role in the effector and regulatory mechanisms of the adaptive immune response. Upon exiting the thymus they begin to undergo a series of phenotypic and functional changes that continue throughout the lifetime and being most pronounced in the elderly. The reason postulated for this is that the dynamic processes of repeated interaction with cognate antigens lead to multiple division cycles involving a high degree of cell differentiation, senescence, restriction of the T-cell receptor (TCR) repertoire, and cell cycle arrest. This cell cycle arrest is associated with the loss of telomere sequences from the ends of chromosomes. Telomere length is reduced at each cell cycle, and critically short telomeres recruit components of the DNA repair machinery and trigger replicative senescence or apoptosis. Repetitively stimulated T-cells become refractory to telomerase induction, suffer telomere erosion and enter replicative senescence. The latter is characterized by the accumulation of highly differentiated T-cells with new acquired functional capabilities, which can be caused by aberrant expression of genes normally suppressed by epigenetic mechanisms in CD4+ or CD8+ T-cells. Age-dependent demethylation and overexpression of genes normally suppressed by DNA methylation have been demonstrated in senescent subsets of T-lymphocytes. Thus, T-cells, principally CD4+CD28^null^ T-cells, aberrantly express genes, including those of the KIR gene family and cytotoxic proteins such as perforin, and overexpress CD70, IFN-γ, LFA-1 and others. In summary, owing to a lifetime of exposure to and proliferation against a variety of pathogens, highly differentiated T-cells suffer molecular modifications that alter their cellular homeostasis mechanisms.

## INTRODUCTION

Immunosenescence comprises a set of changes occurring to the innate and adaptive immune responses that accompany human aging. These result in complex manifestations of still poorly defined deficiencies in the elderly population. Aging is associated with a decline in immune function, with an associated higher susceptibility to infections, incidence of cancer and autoimmunity [1-4]. Aging is also related to poor response to vaccination, including that to the influenza vaccine [5, 6]. At present, individuals can live up to 80-100 years, a much longer time than our ancestors typically managed. Thus, the human immune system has to cope with a lifelong exposure to a variety of antigens that form the basis of immunosenescence, and for which it has not evolved, with a consequent increase in morbidity and mortality due to infections and age-related pathologies [7, 8]. Cellular immunosenescence is characteristic not only of physiological aging but also of some pathologies in which immune activation and inflammation become generalized. In this way, some autoimmune diseases, such as rheumatoid arthritis [9], and some infectious diseases, such as HIV infection [10], are characterized by an accelerated immune senescent phenotype. All these situations cause a state of maintained inflammation, which is thought to be one of the basic processes that leads to the aging of the immune system [11, 12].

The aging process seems to alter both branches of the immune system, the innate and the adaptive, in different ways. While the adaptive immune response undergoes profound age-dependent modifications [13], innate immunity has been considered to be better preserved globally [14, 15]. However, recent data show that aging is associated with chronic innate immune activation and significant changes in monocyte function [16].

Thymocyte progenitor cells go to the thymus and differentiate into CD4+ or CD8+ naïve T-cells, which are then exported to the periphery. This process is regulated by cytokines and hormones as well as by epithelial and dendritic cells, and macrophages and fibroblasts situated in the thymic stroma. In humans, nothing is known about the absolute cell count of recent thymic emigrants (RTEs) although a dramatic decrease of them with age is observed [17]. 

Immunosenescence is not accompanied by an unavoidable and progressive deterioration of the immune function, but is rather the result of a remodeling in which some functions are reduced, others remain unchanged or are even increased [18, 19]. Two major features that T-cells acquire as they age are the loss of proliferative capacity and the acquisition of markers typical of NK cells. These two disorders can be caused by: telomere shortening, T-cell signal transduction changes, alterations in the interaction of the innate and adaptive response, impaired DNA repair and antioxidant mechanisms, epigenetic changes, and persistent antigenic stimulation [20, 21]. In this review we will consider these molecular mechanisms that are so intimately related with the process of immunesenescence and therefore with the poor immune response associated with this phenomenon.

## AGING AND THYMIC INVOLUTION

The thymus is a primary lymphoid organ that plays a crucial role in the development of T-lymphocytes by providing a suitable microenvironment in which these cells can proliferate, for rearranging the TCR and for T-cell maturation, in order to mount an adequate immune response against pathogens and tumors. Throughout life it provides a continuous supply of naïve T-cells, although shortly after the start of youth, the volume of production of the thymus begins to shrink and as a result, increasingly fewer naïve T-cells exit to the periphery [22]. This phenomenon is called “thymic involution” and so far has no satisfactory explanation for its existence [23]. Several hypotheses have argued that this age-related change is adaptive rather than detrimental [24-26]. Accordingly, thymic involution may represent a mechanism for how the body is able to achieve the remarkable balancing act of avoiding autoimmunity and maintaining a sufficiently diverse repertoire to combat a large number of potential pathogens. Some possible causes of thymic involution may be the blocking of the rearrangement of TCR genes [27], self-peptide MHC-decreased molecules [28], and loss of T-cell progenitors [29]. Several authors have shown that, despite thymic involution, new cells migrate from the thymus in adults. This has been demonstrated by direct observation of thymocytes in adult thymus tissue and by quantification of TCR excision circles (TRECs) in peripheral blood [30, 31]. Both naïve CD4+ and CD8+ T-lymphocytes arise from the thymus, but can also remain as a result of expansion of T-lymphocytes found in the periphery. The mechanism by which this peripheral expansion occurs are unclear, but it is postulated that IL-7, IL-15 and other cytokines can stimulate T-cell division without losing their naïve phenotype [32-34]. The behavior of naïve T-cells is different in CD4+ and CD8+ T-cells, whereby the population of naïve CD4+ T-cells modestly declines with age, the numbers of naïve CD8+ T-cells plummet from 65-70 years of age [30, 35]. This suggests that, in the absence of significant thymic influx, the compartment of naïve CD4+ T-cells is sufficiently sustained for another two to three decades by homeostatic proliferation. The mechanism of this rapid decline in naïve CD8+ T-lymphocytes is not known, but, in principle, thymic involution should affect CD4+ and CD8+ T-cells equally. Several studies have proposed that different growth factors and cytokines may be involved in regulating the two distinct populations of lymphocytes [36, 37]. Moreover, phenotypic changes attributed to replicative stress, such as the loss of expression of the CD28 molecule, are more frequently found in the CD8+ than in the CD4+ T-cells. These results all suggest that the homeostasis to which CD4+ T-cells are subjected is much stricter than that experienced by CD8+ T-lymphocytes, possibly because the correct functioning of CD4+ T-cells is more important for the maintenance of perfect immunity, given that these are key cells in almost all immunological pathways.

Thymic functionality could be quantified indirectly by measuring phenotypically naïve T-cells in the periphery or by chest computed tomography (CT) measurement of thymic volume. The first method cannot discriminate long-lived naïve T-cells, and the second is based on the assumption that thymic tissue volume is correlated with functionality. In 1998 Douek and colleagues first reported the use of the TCR excision circles (TRECs), byproducts of the rearrangements that occur during formation of the TCR, to study changes in the frequency of RTEs with age and in the case of HIV infection [30]. TRECs are circular, episomal DNA molecules present in the nucleus, generated during the intrathymic rearrangement of the α- Fig. (**1**) and β-chain Fig. (**2**) loci of the TCR. The removal of genes from the TCR chains does not imply their elimination, as such DNA remains in the nucleus as a circle that cannot replicate. So, when a cell divides, TRECs are passed only to one of the two daughter cells. During subsequent cell cycles, TRECs are then diluted in the population that originates from the first cell [38, 39]. The levels of these DNA molecules in peripheral lymphocytes are a reflection of thymic activity and have been suggested to be the best markers of thymic function [40]. TREC measure ments have been presented in a number of ways. As TRECs are stable molecules that are only diluted during cell proliferation, the sjTREC, a unique molecule generated in most differentiating thymocytes, has been used for several years as a surrogate marker for thymic function. Nevertheless, the profound effect of peripheral proliferation on naïve T-cells in sjTREC counts was quickly noted [41, 42], so, other more reliable methods have had to be investigated. An intense proliferation occurs during lymphocyte differentiation at the thymus, as DJβTRECs are generated before this proliferation, the frequency of DJβTRECs in double-positive (DP) thymocytes is inversely proportional to the number of division cycles these cells have undergone during their differentiation. Moreover, because the sjTREC molecule is produced after this extensive proliferation stage, they are not diluted by this intrathymic cell division. The extent of intrathymic precursor T-cell proliferation, which is proportional to thymic output, can be estimated by quantifying the sjTREC and DJβTREC frequencies and calculating the sj/βTREC ratio measured in peripheral blood mononuclear cells [43]. However, quanti fying the sj/β-TREC ratio is difficult and time-consuming bcecause 11 multiplex PCR reactions with a nested PCR strategy are needed for each reaction. Probably because of these limitations, this procedure has not been used often. A recent study presented a simplified protocol that reduced the amount of PCR reaction needed, while maintaining the sensitivity and reproducibility of the original technique [44, 45]. Its authors designed a nested, multiplex PCR procedure in which only two different PCR reactions are required for the first round of PCR: one amplifies the sjTREC and the other one of the six DβJβTRECs, and the sj/β-TREC ratio is obained from the same PCR reaction. The different methods and units used to measure TRECs affect the interpretation and comparison of the TREC data. Moreover, T-cell proliferation, T-cell death and intra-cellular TREC degradation influence the different assays in distinct ways.

Aging is associated with a situation of immunodeficiency possibly due to the status of thymic involution, amongst other things [46]. The level of TRECs in T-cells of the elderly is much lower than that in young people, due to both the division suffered in peripheral T-lymphocytes and the reduction of the thymic activity associated with thymic involution. Quantification of TRECs has shown that these molecules decrease in lymphocytes in an age-related manner [47, 48]. The use of TREC quantification is useful not only in the study of T-cells in physiological aging, but also in other processes related to immune senescence, such as HIV infection and autoimmune pathologies. Several studies have shown that T-cells in HIV-infected subjects have a lower TREC content than age-matched, uninfected individuals [41, 49]. In auto immunity, T-cells respond to antigenic challenge with a clonal burst, therefore reducing the peripheral levels of TRECs [50]. Lower TREC levels in the peripheral blood of patients with active systemic lupus erythematosus (SLE) but not in those with the quiescent form have also been described [51].

The importance of the thymus for developing adequate cellular immunity can be studied in the context of several disease states (associated with thymic ablation or hypop lasia). It is important to distinguish between individuals who have undergone thymic aplasia or hypoplasia and not generated any new naïve cells (such as DiGeorge, Wiskott-Aldrich and Down syndromes, and severe combined immunodeficiency), and individuals who have had a normal fetal development with initial production of naïve T-cells, but who subsequently suffered a complete or partial thymus ablation, such as occurs in congenital heart disease (CHD). The pathologies of individuals in the first group are related to pre-natal thymic hypoplasia and alterations in the T-lymphocyte population associated with the immuno deficiency generated. However, these patho logies are very complex and we cannot conclude directly from them the importance of the thymus and its role in immunesenescence, in contrast to thymectomized individuals (CHD). Young people who were thymectomized within 2 weeks of birth display several immunological alterations, including lower CD4+ or CD8+ T-cell counts, reduced proportions of recent thymic emigrants and naïve cells, accumulation of oligoclonal memory T-cell populations, and increased levels of inflammation markers [52]. These results indicate that these individuals have premature signs of immune aging and that the phenomenon of immunosenescence is strongly related to the lack of thymic activity and inadequate production of new T-cells.

Although it is well established that the thymus atrophies in the elderly, there is increasing evidence that thymopoiesis continues in the elderly and that involution can be therapeutically reduced or reversed. Some cytokines and growth factors, such as keratinocyte growth factor (KGF) [53], and IL-7 [54], have stimulating effects on the thymus of aged mice. Both molecules have been applied to aged mice in an attempt to reverse the process of thymic involution with varying results. Another possible point of control of thymic aging is the sex steroids, since these hormones are clearly linked to thymic degeneration [55], and intervening in this pathway is a potential means of reversing thymic atrophy. Moreover, it is very important to develop new strategies that enhance thymic functionality and promote immune reconstitution, especially in the elderly. Overall, research into and knowledge of the mechanisms leading to age-related thymic involution are essential if we are to develop new strategies to combat age-related immunosenescence and to maintain an effective immune response throughout life. 

## TELOMERES AND T-CELL IMMUNOSENESCENCE

A defining feature of the eukaryotic genome is the presence of linear chromosomes. This arrangement, however, poses several challenges with regard to chromosomal replication and maintenance. Telomeric DNA is lost due to the incomplete terminal synthesis of the lagging DNA strand during cell division [56]. Immune cells must be able to grow exponentially and die when no longer needed. They support an extremely high replicative rate, so their telomeres suffer great stress. The lymphocytes are capable of upregulating telomerase, an enzyme that elongates telomeres and can therefore prolong the life of the cell [57, 58]. In the absence of mechanisms that compensate for telomere shortening, growth arrest of the cell occurs when progressive telomere erosion reaches a critical point known as replicative senescence [59, 60] Fig. (**3A**). Telomere length can be estimated from the terminal restriction fragment (TRF), which contains the telomere TTAGGG region and some nontelomeric sequences. TRF length varies among the cell populations studied and even in the chromosomes within the same cell, so cellular senescence could be achieved when TRF reaches a critical size that has been estimated as less than 6 Kb [61] Fig. (**3B**). The overall finding from several different studies is that human T-cells can undergo a limited number of divisions, after which they cease dividing [62, 63]. Importantly, the arrival of T-cells at a stage of replicative senescence does not imply the loss of cell viability. In fact, under appropriate conditions senescent-cells remain alive and metabolically active for a long period of time [64, 65]. Germline cells, many malignant tumor cells, and certain stem cells do not undergo replicative senescence, and have stable chromosome lengths despite extensive replication, due to the constitutive activity of telomerase. 

To study differentiation-related changes in telomerase activity, it is important to discriminate between undifferentiated and highly differentiated T-cell populations. T-cells can be separated into functionally different populations using combinations of cell surface markers such as the tyrosine phosphatase isoform CD45RA and the chemokine receptor CCR7. With these markers, we subdivided the T-cells into naïve cells (NAÏVE; CD45RA+CCR7+), which have not yet contacted their antigen, as is necessary to initiate the immune response; central memory cells (CM; CD45RA-CCR7+), which have already contacted their antigen and retain a high proliferative capacity; effector memory cells (EM; CD45RA-CCR7-), which are highly differentiated and produce various factors that help maintain the immune response; and effector memory RA cells (EMRA; CD45RA+CCR7-), which form a highly differentiated population that has lost some of its functional capacity [66] Fig. (**4A**). EM and EMRA are heterogeneous populations, and the staining of two additional markers, CD27 and CD28, has proved useful for identifying less differentiated (CD27+ and/or CD28+) or more differentiated (CD27^null^CD28^null^) cells [67] Fig. (**4B**). Differentiating CD4+ T-cells lose expression of CD27 first and subsequently of CD28 at a later phase [68, 69]. In contrast, CD8+ T-cells lose expression of CD28 first and then of CD27 [70]. The relatively undifferentiated T-cells (CD27+CD28+) have longer telomeres than most differentiated T-cells (CD27-CD28-), and intermediate populations (CD27-CD28+ in CD4+ T-cells and CD27+CD28- in CD8+ T-cells) have telomere lengths between those of the undifferentiated and highly differentiated cells [71, 72]. Furthermore, several studies have shown that the ratio of proliferation in human T-lymphocytes is much higher in highly differentiated cells CCR7-CD27-CD28- with shorter telomeres [73]. Telomerase activity is proportional to the length of telomeres, in that it is higher in undifferentiated populations and much lower in the more differentiated populations, and the ability to induce this enzyme is lost as cells age [71, 72, 74]. In addition to these differences in lymphocyte subpopulations they changes in the telomeric behavior have been described between CD4+ and CD8+ T-cells. In cultures where CD4+ and CD8+ T-cells of the same person are subjected to identical stimuli, CD8+ T-cells were unable to upregulate telomerase after the fourth encounter with the antigen. In contrast, the CD4+ T-cells from the same donor had a high level of telomerase activity induced by antigen [75]. Previous studies have shown that the homeostasis to which the population of CD4+ T-cells is subject is much stricter than that suffered by the population of CD8+ T-cells. In fact, the aging of lymphocyte populations was described primarily in CD8+ T-lym phocytes, where these changes occur more precociously [76, 77].

Telomere loss throughout life could potentially be due to the repeated activation of specific T-cells or to cumulative oxidative damage [39]. This hypothesis is supported by the fact that telomere shortening has been observed in diseases featuring chronic contact with antigens and, therefore, in which there is chronic activation of the lymphocyte population [78]. This is the case of HIV infection [79], rheumatoid arthritis [80], psoriasis, and atopic dermatitis [81]. Oxidative stress accelerates telomere shortening in cell culture. Short-term exposure of T-lymphocytes to oxidized LDL drives them to replicative senescence and stimulates the production of NFkB [82]. In addition to viral infections and oxidative damage, there are other types of stress that compromise immune response, such as dementia family caregivers or parents of chronically ill children [83, 84]. Chronic psychological stress is also associated with telomere shortening and reduced telomerase activity [85, 86]. The hormone cortisol inhibits telomerase activity in CD4+ and CD8+ T-cells, suggesting a mechanism by which stress can negatively affect immune responses [87]. All these studies suggest that chronic stress can end up in increased oxidative stress, decreased telomerase activity and shortened telomeres.

Signaling via the T-cell receptor (TCR) and other co-stimulatory molecules such as CD28 are necessary for the induction of telomerase activity with a peak of activation at 4-5 days after being stimulated and a decrease in activity at 10 days [72, 88, 89]. T-cells can be induced to proliferate by certain cytokines without a signal via TCR, in a process known as homeostatic proliferation, whereby the naïve and memory cells are maintained by providing survival signals and driving homeostatic proliferation [90]. IL-7 and IL-15 have been associated with induction of telomerase activity in CD4+ and CD8+ T-cells, respectively, [91, 92]. We have recently demonstrated that IL-15 has a powerful preferential effect on the population CD4+CD28^null ^T-cells, increasing the proliferation and specific immune response of these cells [93]. There was also an inhibitory effect on telomerase activity of certain cytokines such as IFN-α or TGF-β [94, 95].

However, what drives the generation of senescent T-lymphocytes *in vivo*? It has been suggested that latent infections with several herpes viruses, which are endemic and persist throughout life in infected individuals, are the main culprits, and constant and prolonged T-lymphocyte activity, involving proliferation, may drive certain virus-specific T-cells to senescence [96]. Several cross-sectional studies of humans have demonstrated that the main accepted age-associated changes to parameters used to assess adaptive immune status are markedly influenced by cytomegalovirus (CMV) infection. The accumulation of late-differentiated T-cells lacking CD28 is well documented in several populations infected by CMV [97, 98]. It has often been stated that this type of late differentiated T-cell is senescent, based on CD28 negativity, expression of CD57, possession of short telomeres, refractoriness to apoptosis and poor proliferative capacity. These effector cells may remain present in order to keep control of CMV and prevent disease. A compensatory increase in these cells seen during aging, may suggest the increasing pressure on the immune system to maintain immunosurveillance against CMV throughout the long human lifespan [99]. Recently, we observed that the elderly with worse functional capacity had a higher anti-CMV titer and T-cell response to CMV than the elderly with better functional status, and demonstrated a relationship between the intensity of the response to CMV, immune system status, and the functional ability of older people [47].

One of the most promising applications of the study of telomeres would be to restore telomerase activity of T-cells and to try to reverse the shortening of telomeres, given the multiple deleterious effects of senescent T-cells. Several studies have shown that telomerase activity is preserved and replicative senescence is delayed if telomere length is stabilized [87, 100]. The inhibition of cytokines involved in shortening of telomeres, such as TNF-α, could delay telomeric loss. Alternatively, given that one of the main factors involved in immune senescence is CMV infection, a preventive vaccination against this virus early in life would be suitable.

## IMPACT OF AGE ON T-CELL SIGNALING

In addition to the loss of telomerase activity and thymic involution, defects in the intracellular signaling of T-lymphocytes have been postulated as being another possible cause of malfunction in the process of immunesenescence. Failures in signaling could lead to changes in the type, intensity and duration of the immune response, all contri buting to the process of immunesenescence. 

Activation of T-cells is a process comprising several stages. First of all, the polymorphic TCR binds together with CD4 or CD8 coreceptors, with the major histocompatibility complex type I (CD8) or type II (CD4 ) attached to its corresponding antigen on the surface of antigen presenting cells (APCs). This contact is the first essential signal in the lymphocyte activation process [101, 102]. The TCR complex has long intracytoplasmic tails that transmit the signal to other molecules through its immunoreceptor tyrosine activation motifs (ITAMs). After the TCR binds with the MHC, several ITAMs are phosphorylated, and ensuring that a large number of kinases, adapter molecules and signaling intermediates are recruited [103]. Antigen recognition is not enough to trigger an immune response, so a second signal is required for lymphocyte activation. These signals are produced by co-stimulatory molecules, the best studied of which is CD28, [104, 105]. Binding of CD28 to its co-receptor molecule (B7) allows the threshold of T-cell activation to be lowered and produces an amplifying effect of the first signal produced by the binding of TCR and MHC molecules. Thus, TCR-MHC binding and CD28-B7 can cause activation of T-lymphocytes [106]. A curious aspect of this is that CD4+ T-lymphocyte immune synapses are much more durable than these of CD8+ T-cells [107]. This probably reflects the different functions served by both types of lymphocytes: CD8+ T-lymphocytes interact with their target to kill them, requiring only a short immunological synapse. However, the pivotal role of CD4+ T-cells, directing the immune response, requires longer contact. The intensity and duration of the contact between T cells and APCs, as well as the microenvironment pro- or anti-inflammatory, means that the lymphocyte response is highly variable. The interaction of TCR and CD28 receptors leads to the production of cytokine, one of the best studied of which is IL-2, which is produced in an autocrine form and that causes upregulation of its own receptor (IL-2R), which is composed of three subunits (α, β and γ) [108, 109]. The γ-chain is common in other cytokines such as IL-7, IL-15 and IL-21, and the differences in the responses elicited by these two cytokines must lie in the other two chains that form the receptor. The main molecules involved in signaling via IL-2 are Janus kinases (Jaks) and signal transducer and activator of transcription (STATs) [110].

One of the first indications that the immune system of the elderly has impaired functionality was the reduction in the production of IL-2 [111]. Several studies have demonstrated that levels of TCR in T-cells are not altered in the elderly, for which reason it is thought that the problem may be to do with intracellular signaling [112, 113]. Alterations in intracellular signaling may partly explain the lack of production of IL-2 in the elderly. However, although the TCR appears not to be altered in the elderly, co-stimulatory molecules required for lymphocyte activation appear to be altered in the elderly. One of the major co-stimulatory molecules present in T-lymphocytes is the CD28 molecule. The loss of this molecule as individuals age is well-documented in CD4+ [114] and CD8+ T-cells [115]. The loss of this molecule has been associated with the process of immunesenescence, and therefore, with a loss of immune system responsiveness in the elderly. It has been reported that these cells are less able to proliferate than are CD28+ T-lymphocytes, have a diminished antigenic recognition repertoire, and gain a very powerful cytotoxic capacity [88, 116]. The loss of the molecule CD28 on T-cells occurs after persistent antigenic stimulation, and after each cycle of stimulation/proliferation, CD28 expression decreases on the surface of T-cells, producing an accumulation of highly differentiated T-cells [117]. The activation and differentiation-induced loss of CD28 is supported by the observations that CD28^null^ T cells have shorter telomeres than their CD28+ counterparts, even when studied in the same clonal population [118]. CD28 downregulation with T-cell activation involves transcriptional repression and increased protein turnover and is thought to be a negative feedback mechanism [119]. When T-cells recognize an antigen, CD28 expression decreases rapidly, but immediately returns to normal levels. However, with sustained stimulation over time, the expression of CD28 decreases and may be lost. CD28 can be initially reinduced by IL-12 [120], or with treatment with anti-TNF agents [121], but once firmly established, CD28 loss is irreversible in the majority of CD28^null^ T-cells, suggesting active transcriptional silencing. Studies using the minimal CD28 promoter have shown the transcriptional activation to be defective in CD28^null ^T-cells. This defect has been associated with a unique transcriptional initiator element (INR) in the CD28 promoter, which consists of two nonoverlapping α and β motifs that have distinct protein-binding profiles but function as a unit. The basal transcription of the CD28 gene is regulated by these two sequences situated downstream from an atypical TATA box. Mutation or deletion of either motif is sufficient to inactivate the CD28 gene promotor. In CD28^null ^T-cells, the αβ-INR is inoperative because of the coordinated lack of sites for α- and β-specific transcriptions factors. These include general transcription factors such as transcription factor II-I and the components of transcription factor IID, or regulatory proteins such as YY1 and USF [122, 123]. However, the CD28 αβ-INR has no homology with other INRs, so there are specific INR-binding proteins including nucleolin and the A isoform of heterogeneous ribonucleoprotein (hnRNP-D0A), which are two ubiquitous mammalian proteins [124]. Therefore, there is evidence to show that INR-regulated transcription is independent of the known components of the basal transcription complex. DNA binding complexes are rapidly lost in proliferating CD8 T-cells while they persist in CD4 T-cells, which might explain the resistance of CD4 T-cells to losing the CD28 molecule with age.

Although CD28 is a major co-stimulatory molecule, these CD28^null^ T-cells remain functionally active; other molecules must be able to maintain responsiveness and survival in these cells. Therefore, alternative receptors must exist to prevent these cells entering into a state of anergy. To date, several co-stimulatory molecules have been postulated as possible alternatives to CD28, including CD27 and 4-1BBL [125, 126]. The involvement of CD27 in lymphocyte activation would make sense in CD8+ T-cells because these cells lose the CD28 molecule first and then CD27 [70]. However, in CD4+ T-lymphocytes exactly the opposite occurs, and they lose the CD27 molecule ifrst, which makes it is impossible that CD27 supplies the function of the CD28 co-stimulatory molecule [68, 69]. As in the case of 4-1BBL, there are very few studies on the subject and we do not know whether this molecule undergoes changes during aging, in the same way that CD28 does.

Another aspect of the intracellular signaling that may be altered in the elderly is the balance between positive and negative signaling, a field in which very little is known so far. The principal molecules involved in the inhibition of signaling are SHP-1/2, CD45 and SHIP1 and co-receptor CTLA-4 and PD-1 [127-129]. As individuals age, not only is there a decrease of the activating molecules, but there is also an increase in the inhibitory process. Clearly, there are many gaps in our knowledge about cell signaling in the aging immune system. Besides T-cells, other cell types that are part of the immune system also undergo age-related changes. There are a few studies in these cells (memory B cells, monocytes/macrophages, dendritic cells, neutrophils, NKs) to help us understand a little better the processes that regulate the signaling pathways in the aging immune system and its functioning in old age. 

## NATURAL KILLER CELL-RELATED RECEPTORS (NKRs) AS BIOMARKERS OF T-CELL AGING

The search for biomarkers of aging is very complicated due to the complexity of the aging process and the processes that accompany it, since it has effects on the development of the various systems and their ability to function. Moreover, a good marker of aging must change as the organism ages. The notion that biomarkers that are predictive of longevity in early life may not be predictive in late life and vice versa leads to quite different strategies in biomarker research. 

Hence, we discuss some lymphocyte-related biomarkers, which are useful for a better understanding of aging, based on immunosenescence, inflammatory responses and oxidative stress. Exposure of T-lymphocytes to pathogens throughout life leads to a decrease in the pool of naïve T-lymphocytes and an increase in memory T-cells with a diminished TCR repertoire produced by an exponential increase of oligoclonal T-lymphocytes. However, although oligoclonal senescent CD28^null^ T-cells are functionally active [117] they are neither anergic nor apoptosis-prone. CD28^null^ T-cells are resistant to apoptosis [130], which is one possible cause of its accumulation throughout life [131]. However, its function seems to depend less and less on the stimulation via TCR [88]. A plausible explanation of why these cells are able to be activated, despite their lack of CD28 molecules, is the *de novo* expression of several natural killer (NK) cell-related receptors (NKRs) [132]. Among the best studied are the receptors CD16, CD56, CD94, KLRG1, several members of the NK receptor G2 (NKG2), and the killer cell immunoglobulin (Ig)-like receptor (KIR) families. The expression of these NK molecules is associated with increased cytotoxic capacity with high levels of expression of intra cytoplasmic perforin and granzyme, but diminished proli ferative capacity and defective production of IL-2 [133, 134]. The expression of these NK receptors in T-lymphocytes probably serves to regulate the cytotoxicity of these cells and even cytokines implicated in NK cell activation, such as IL-15, are able to enhance their cytotoxic ability. The expansion of these cells not only appears in the elderly, but also in other clinical conditions involving chronic activation of the immune system, such as viral infections, autoimmune and rheumatic diseases, certain tumors and coronary artery disease [135-137] Fig. (**5**). In the case of artery disease and CMV infection, the expression of KIR receptors in CD4+CD28^null^ T cells is broadly accepted to be responsible for their functionality [138, 139]. Meanwhile, progression of the rheumatic diseases is thought to be accompanied by the recruitment and rise of oligoclonal, autoreactive CD4+CD28null T cells, present a low activation threshold in response to TCR stimulation, which could be implicated in its predisposition to the breakdown of self-tolerance [140].

CD94, KLRG1 and the NKG2s are lectin-like receptors, and CD16 and CD56 are receptors belonging to the superfamily of immunoglobulins, and are the prototypic NKRs that are normally used to identify NK cells. The functional roles of CD16, CD56 and CD94 on senescent T-cells are still unknown. KLRG1 receptor seems to influence the state of T-cell senescence due to their ability to inhibit proliferation via TCR [141, 142]. KLRG1 contains an immunoreceptor tyrosine-based inhibitory motif (ITIM) in its cytoplasmic domain and has been shown to be a receptor for some members of cadherin family of proteins [143]. It is an inhibitory receptor and its presence in T-cells blocks the co-stimulatory activities mediated by Akt, such as proliferation [144]. Among NKG2s receptors, only NKG2D has been shown to express in CD28^null^ aged T-cell, increasing its expression in CD8+ T-cells in the elderly [145] and its expression being newly present in CD4+CD28^null^ T-lymphocytes as people age. This novel age-marker was recently described by our laboratory [146]^146^. This molecule has been implicated in NK-mediated anti-viral immunity and in TCR-independent cytotoxic activity in CD4+ and CD8+ T-cells. The regulation of KIRs seems to differ in NK cells and T-lymphocytes [147]. The KIR repertoire in T-cells is very restricted [132], being limited to memory T-cells, mainly CD28^null^ T-lymphocytes. In addition, the same population of T-lymphocytes with the same TCR specificity may have different combinations of KIRs on its surface [138, 148]. It seems quite clear that there is a different expression of NKRs in oligoclonal and senescent T-cells. The expression of these molecules appears to represent a different way of diversifying the immune repertoire, i.e., an oligoclonal population of T-lymphocytes for a particular TCR can express a wide diversity of receptors NKRs codominantly [133, 149]. The appearance of these molecules in senescent T-cells could help maintain the adequate homeostasis of T-cells and would be a way to stay functionally active independent of TCR activation. A co-stimulus produced by any of these molecules could activate the cells with a functional TCR without the need for CD28 co-stimulation. 

In summary, compared with young people, the elderly have a much less robust immune response, but there is wide phenotypic diversity among the elderly, who range from being fully dependent to having a completely normal and independent life [150, 151]. Despite a history of chronic conditions, some elders neither exhibit significant physical nor cognitive disability nor do they become frail, a phenomenon referred to as exceptional aging [152]. One possible approach to try to improve immunity in elders is to identify cellular and humoral parameters that are involved in immune competence or incompetence, such as NKRs and inflammatory pathways, as well as predicting future health outcomes. 

## EPIGENETIC AND IMMUNOSENESCENCE

The relationship between aging and epigenetic changes occuring throughout the lifetimes of organisms is beoming increasingly clear [153, 154]. These changes are perpetuated by the replication of DNA and are transmitted to daughter cells. Many of the processes that are triggered during cell differentiation are regulated by epigenetic changes. These epigenetic processes include DNA methylation by methyltransferases (Dnmts), histone modifications such as methylation, acetylation and phosphorylation, structural modifications of chromatin, and microRNAs and other noncoding regulatory RNA [155, 156]. Hypomethylation in regions rich in CpG islands and acetylated histones produces active transcription, whereas DNA hypermethylation and histone hypoacetylation reduces gene transcription. The epigenetic pattern of an individual is mainly established during intra-uterine life, but epigenetic changes also occur during extra-uterine life. Such changes may occur at a rate one or two orders of magnitude greater than that of somatic mutations. Many of these changes during adulthood may arise from changes in the environment surrounding the individual, as can be deduced from some twin studies that claim that the less time twins have shared together, the greater the differences are in their epigenetic DNA. On the other hand, it has been shown that as the body ages, methylation decreases in repeated regions of the human genome [157]. It is postulated that the decrease in Dnmt1 activity is one of the main cause of this reduction in the methylation in aged organisms [158, 159]. 

Apart from individual variation in genetic predisposition, epigenetic changes over the full course of human life exert immunomodulatory effects. Most data about changes in DNA methylation in aging has been obtained from epithelial cells, but several studies have examined these changes in the immune system. Global hypomethylation has been reported in the thymus of aged cows [160] and in T-lymphocytes of elderly humans [161], but more work is needed to identify the genes involved. The availability of dietary precursors of methyl donors like folate, choline and methionine is very important, so a diet that does not provide these precursors could influence methylation patterns. Low levels of these precursors at an early age results in an increase in life expectancy in mice as well as a preservation of immune function [162], which could be related to the increased length of life in animals subjected to a hypocaloric diet. Inhibition of DNA methylation increases the expression of lymphocyte function-associate antigen-1 (LFA-1), involved in T-cell activation. This increase can be observed in the elderly and in patients with systemic lupus erythematosus (SLE) [163]. The acetylation of histone H4 and H3 phosphoacetylation followed by pro-inflammatory signals raises NF-κB activity and consequently increases levels of IL-6, which increase with age [164, 165]. Treatment with phytohaemagglutinin (PHA)-stimulated peripheral blood lymphocytes with an inhibitor of histone deacetylase (HDAC) causes hypomethylation of histone H4 that increases with age. Mice treated in this way produce raised levels of Foxp3+CD4+CD25+ (Treg) cells [166]. The mechanisms that drive increasing NKRs expression in T-cells with age are unclear. NKRs are primarily expressed in natural killer (NK) cells, where they are clonally distributed. Inhibition of the DNA methyltransferase (DNMT) by 5-aza-2’-deoxycytidine (5-Aza-dC) leads to the global expression of KIRs in all NK cells [167]. Several studies have shown that KIR transcriptional control differs in T-cells and NKs: even naïve CD4+ T-cells have the transcriptional machinery to support the activation of the minimal KIR promoter in reporter gene assays [147]. Several studies report that some of the genes overexpressed in senescent T-cells, such as CD11a, perforin, CD70, IFN-γ and the NKRs family, are normally suppressed in T-cells by DNA methylation and that inhibiting replication of DNA methylation patterns during mitosis is sufficient to increase their expression [168-170]. Other study demonstrates that the CD70, perforin, and KIR2DL4 promoters are demethylated in CD4+CD28^null^ T-cells, that Dnmt1 and Dnmt3a levels are decreased in this subset, and that siRNA knockdown of Dnmt2, but not Dnmt3a, causes similar demethylation and overexpression of these molecules [171]. Therefore, epigenetic changes may control the increased frequency of NKRs in T-cells with aging. 

MicroRNAs (miRNAs) consist of short noncoding RNA molecules of approximately 18-22 nucleotides that regulate post-transcriptional gene expression by degradation or repression of mRNA molecules [172]. As the recognition of target mRNAs mainly depends on the small seed region within the mature miRNA, a single miRNA potentially regulates up to several hundred mRNA targets, thus orchestrating a large variety of cellular processes [173]. miRNAs are known to play an important role in the thymic development of T-cells [174]. The miRNA-17-92a cluster regulates the immune system and is essential for lymphoid cellular development and tumorigenesis in lymphoid tissue [175]. Although miR-17-92a expression is crucial for lymphocyte development, there are few reports on *in vivo* human lymphocyte senescence. One such study indicated that the age-related attrition of naïve T-cells is linked to a reduction of miR-92a in human T-lymphocytes [176]. The quantitative polymerase chain reaction has been used to shown demonstrate the age-related downregulation of miR-17, miR-19b, miR-20a and miR-106a, some of which are involved in tumorigenesis and cell cycle control [177]. Briefly, all these results indicate specific changes of miRNA abundance and activity in a broad range of human aging models and suggest that miRNAs could be used as novel biomarkers of cellular aging.

Recently, epigenetic changes and certain miRNAs have been recognized as being part of normal and pathological aging. Epigenetics has allowed us to link the genetic and environmental changes invovled in human diseases. Several drugs are being introduced with epigenetic targets in cancer therapy and other pathologies where these mechanisms have been involved. For all these reasons, the study of epigenetic mechanisms and miRNAs involved in the process of immunosenescence will answer many of the currently unanswered questions. 

## CONCLUSIONS

Aging has very specific effects on T-cell function. The immune response to infections, immunizations and tumors found in the elderly is quite different from that found in young people. This is the result of various factors, including the lack of proliferative capacity, the appearance of NKRs, changes in TCR repertoire and the development of clonal expansions. One possible and promising intervention that could be employed in an attempt to improve the immune response in the elderly is thymic reconstitution, by which we might prevent the loss of naïve T-cells and slow down the accumulation of exhausted and aged CD28^null^ T-cells. Preclinical and clinical studies of T-cell reconstitution are being undertanken to achieve this purpose of "rejuvenating" the T-lymphocyte population. These studies include molecules such as IL-7, IL-15, IL-12, keratinocyte growth factor (KGF), growth hormone (GH), insulin growth factor-1 (IGF-1) and ghrelin. It would also be interesting to try to regulate epigenetic mechanisms that occur in the extrapolation of T-cells with age, with drugs that control these mechanisms, such as inhibitors of class I and class II HDACs, evaluated especially for cancer therapies and inflammatory diseases. Therefore, further studies will help us to develop new strategies to slow or reverse age-associated immune dysfunction.

## Figures and Tables

**Fig. (1):**
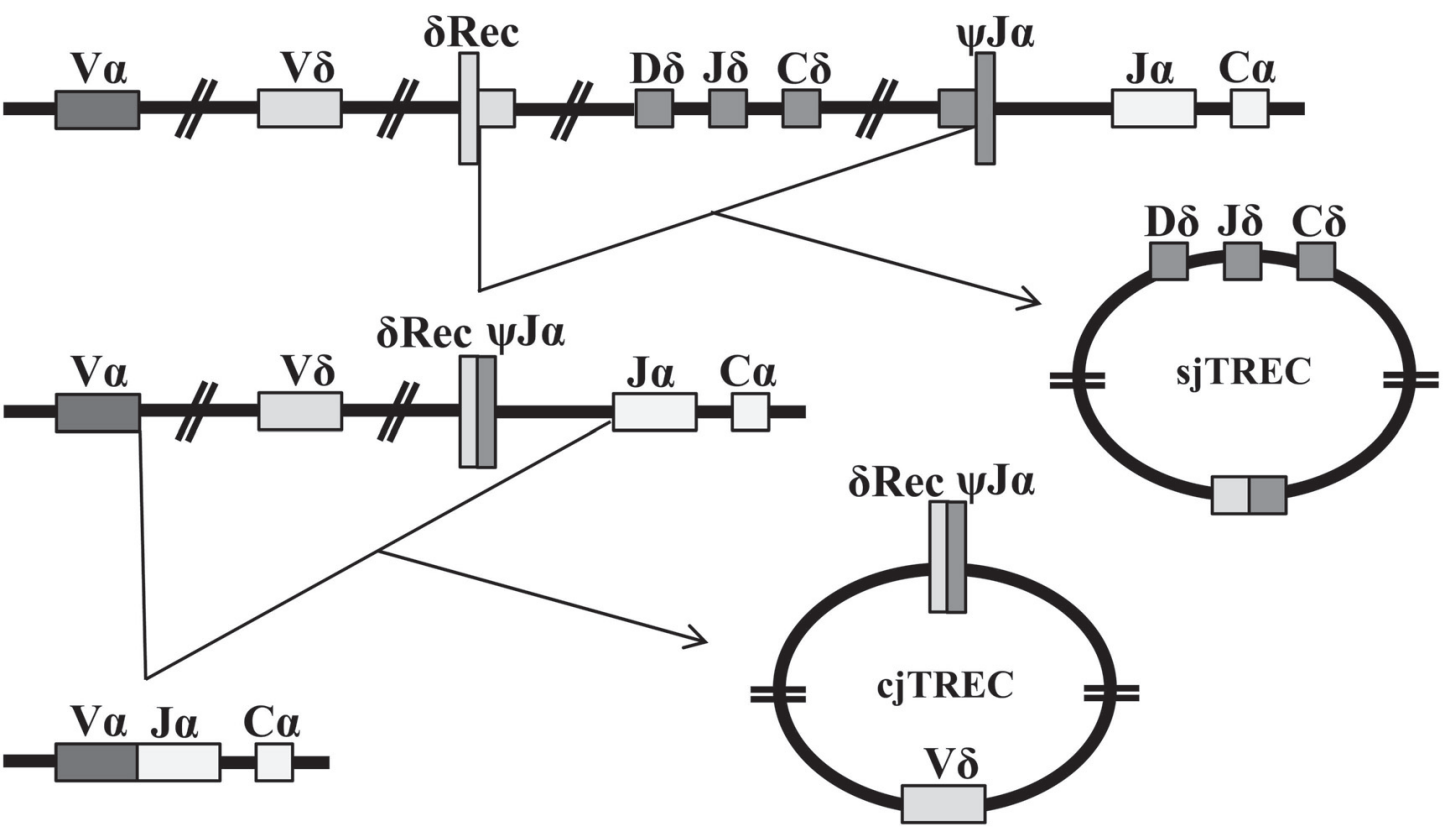
**Formation of TRECs at the TCRα locus.** During their passage through the thymus, T-cell precursors rearrange their T-cell receptor (TCR) genes. The TCRα chain sequence borders the TCRδ locus and, to rearrange a TCRα chain, the double-positive (DP) thymocytes must excise the TCRδ loci, committing the T-cell to the αβ T-cell lineage. Genes for the δ-chain of the TCR are distributed within the genomic region that codes for the α-chain along chromosome 14q11, and are removed in two steps during the recombination of Vα with Jα, this generates the signal-joint TREC (sjTREC) that can be quantified by PCR. Then, thymocytes proliferate three or four times and finally complete the rearrangement of Vα with Jα, thereby producing the second TREC, known as coding-joint TREC (cjTREC).

**Fig. (2):**
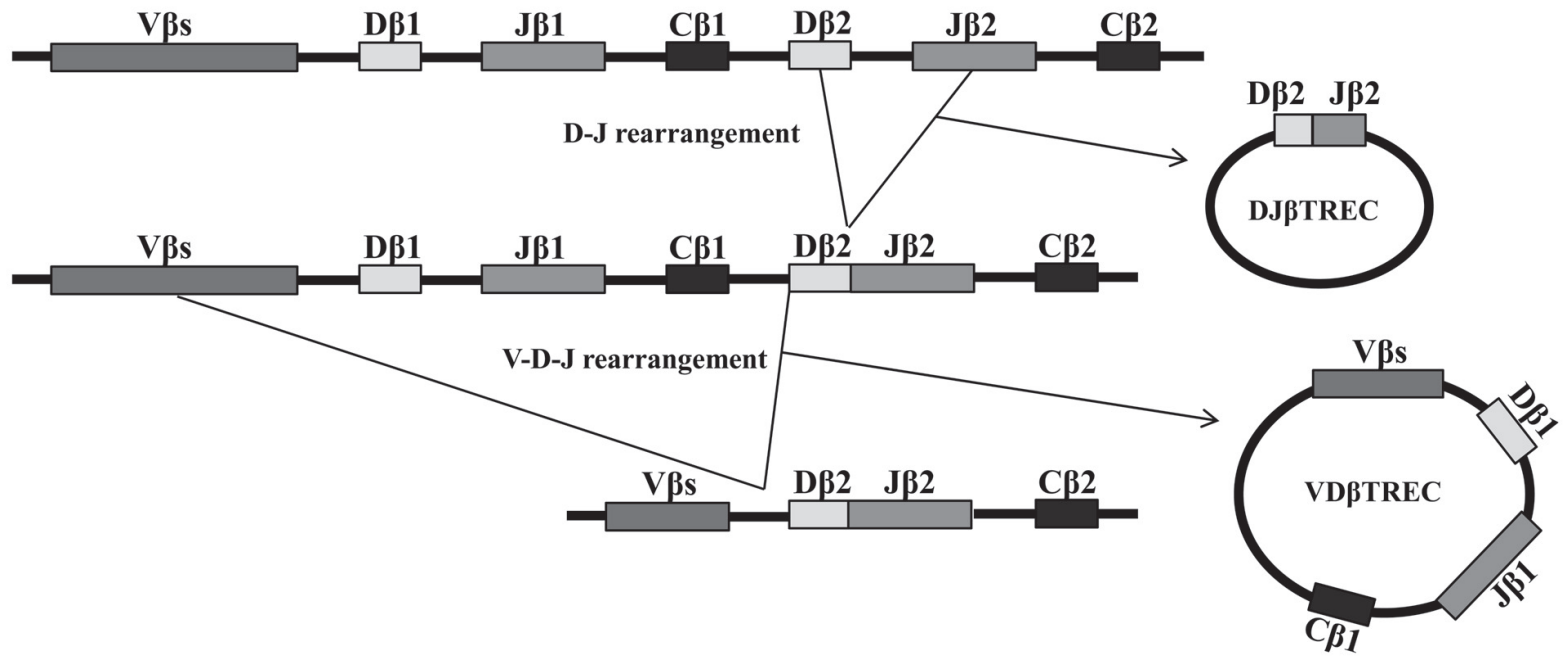
**Schematic diagram of sequential rearrangement steps at the TCRβ locus.** Two successive genetic rearrangements occur at the β- chain. The rearrangement of the TCRβD to TCRβJ segments occurs first and generates a by-product, a specific DJβTREC. This is followed by the recombination of the V to DJ segments, which also generates a specific VDβTREC. These two types of TREC molecules can be quantified, but there are too many possibilities with 65 Vβ segments to consider all rearrangements.

**Fig. (3):**
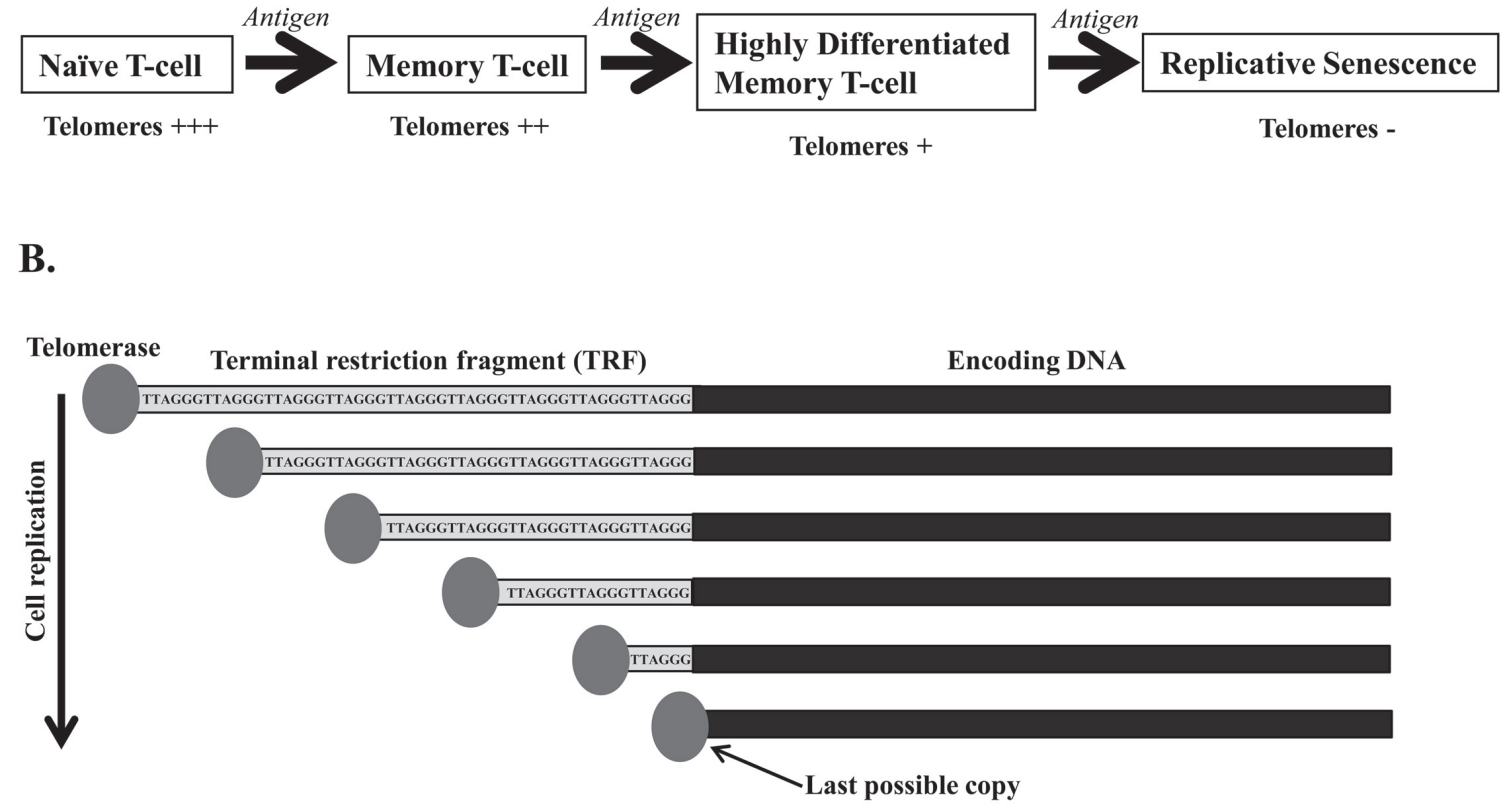
**Schematic representation of replicative senescence and terminal restriction fragment (TRF).** (**A**) After their first encounter with an antigen, naïve T-cells are activated, proliferate and differentiate into memory T-cells. Most of these memory T-cells die by apoptosis, leaving a small pool of memory T-cells that, in subsequent encounters with the antigen, will become highly differentiated memory T-cells. These cells have shorter telomeres and have lost the ability to up-regulate telomerase activity. Subsequent encounters with antigens make these cells enter replicative senescence. (**B**) The TRF contains the telomere TTAGGG region and some nontelomeric sequences. Cellular senescence could be achieved when the TRF reaches a critical size, which has been estimated to be less than 6 Kb.

**Fig. (4):**
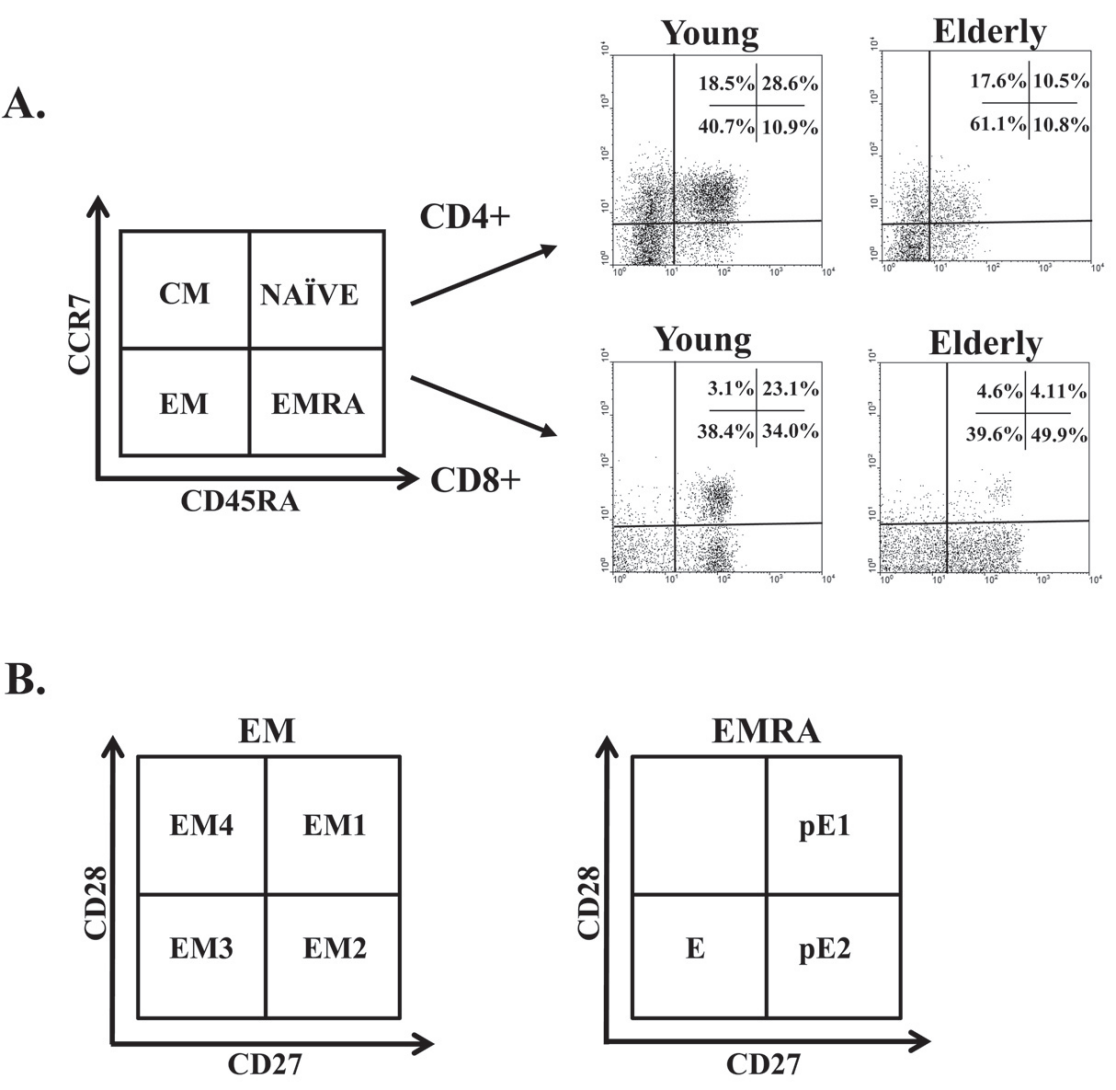
**Distribution of CD4+ and CD8+ T-cells into naïve, central memory, effector memory (EM) and effector memory RA (EMRA) cells, and distribution of EM and EMRA cells into subsets defined by CD28 and CD27 expression.** (**A**) Schematic model of the T-cells differentiation subsets with respect to CD45RA and CCR7 expression and dot plots representative of these subsets in young people and elderly subjects. (**B**) Representative dot plots of the subsets defined by CD27 and CD28 expression for individuals in each group. EM Tcells can be divided into EM1 (CD27+CD28+), EM2 (CD27+CD28^null^, only in CD8+ T-cells), EM3 (CD27^null^CD28^null^) and EM4 (CD27^null^CD28+). Similarly, EMRA cells can be divided into pE1 (CD27+CD28+) and pE2 (CD27+CD28^null^, only in CD8 T-cells) and E (CD27^null^CD28^null^) groups.

**Fig. (5):**
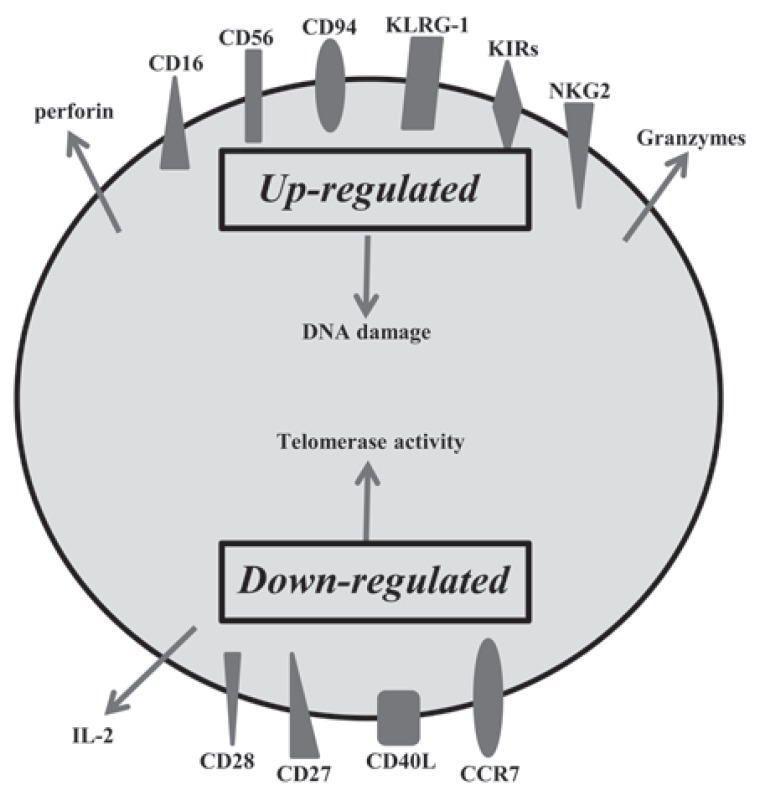
**Summary of changes in exhausted memory T-cell.** Age is associated with several immune changes, especially in T-cell phenotypes. Exhausted T-cells are monoclonal expansions and are specific to a few antigens. These cells lose the ability to home to secondary lymphoid organs, produce pro-inflammatory cytokines and have a high cytotoxic capacity.
